# Psilocybin—Mediated Attenuation of Gamma Band Auditory Steady-State Responses (ASSR) Is Driven by the Intensity of Cognitive and Emotional Domains of Psychedelic Experience

**DOI:** 10.3390/jpm12061004

**Published:** 2022-06-19

**Authors:** Vojtěch Viktorin, Inga Griškova-Bulanova, Aleksandras Voicikas, Dominika Dojčánová, Peter Zach, Anna Bravermanová, Veronika Andrashko, Filip Tylš, Jakub Korčák, Michaela Viktorinová, Vlastimil Koudelka, Kateřina Hájková, Martin Kuchař, Jiří Horáček, Martin Brunovský, Tomáš Páleníček

**Affiliations:** 1 National Institute of Mental Health, Topolová 748, 250 67 Klecany, Czech Republic; vojtech.viktorin@nudz.cz (V.V.); dominika.dojcanova@nudz.cz (D.D.); peter.zach@nudz.cz (P.Z.); anna.bravermanova@nudz.cz (A.B.); veronika.andrashko@nudz.cz (V.A.); filip.tyls@nudz.cz (F.T.); jakub.korcak@nudz.cz (J.K.); michaela.viktorinova@nudz.cz (M.V.); vlastimil.koudelka@nudz.cz (V.K.); jiri.horacek@nudz.cz (J.H.); martin.brunovsky@nudz.cz (M.B.); 2 Third Faculty of Medicine, Charles University, Ruská 2411, 100 00 Prague, Czech Republic; 3 Institute of Biosciences, Vilnius University, 7 Saulėtekio Ave, 10257 Vilnius, Lithuania; aleksandras.voicikas@gmc.vu.lt; 4 First Faculty of Medicine, Charles University, Kateřinská 32, 121 08 Prague, Czech Republic; 5 Forensic Laboratory of Biologically Active Substances, Department of Chemistry of Natural Compounds, University of Chemistry and Technology Prague, Technická 5, 166 28 Prague, Czech Republic; katerina.hajnakova@vscht.cz (K.H.); martin.kuchar@vscht.cz (M.K.)

**Keywords:** psilocybin, psychedelics, auditory steady-state response, model of psychosis, EEG, serotonin

## Abstract

Psilocybin is a classical serotoninergic psychedelic that induces cognitive disruptions similar to psychosis. Gamma activity is affected in psychosis and is tightly related to cognitive processing. The 40 Hz auditory steady-state responses (ASSR) are frequently used as indicators to test the ability to generate gamma activity. Based on previous literature, we studied the impact of psilocybin on 40 Hz ASSR in healthy volunteers. The study was double blind and placebo controlled with a crossover design. A sample of 20 healthy subjects (10M/10F) received psilocybin orally 0.26 mg/kg or placebo. Participants were measured four times in total, one time before ingestion of psilocybin/placebo and one time after ingestion, during the peak of intoxication. A series of 500 ms click trains were used for stimulation. Psilocybin induced a psychedelic effect and decreased 40 Hz ASSR phase-locking index compared to placebo. The extent of the attenuation was related to Cognition and Affect on the Hallucinogen Rating Scale. The current study shows that psilocybin lowers the synchronization level and the amplitude of 40 Hz auditory steady-state responses, which yields further support for the role of gamma oscillations in cognitive processing and its disturbance.

## 1. Introduction

Psilocybin (O-phosphoryl-4-hydroxy-N, N-dimethyltryptamine) can be found in many species of psychoactive fungi and is classified as a classical serotoninergic psychedelic drug, which acts as an agonist at serotonin 5-HT2A/C and 5-HT1A receptors [1]. Psilocybin has recently received a great deal of attention as a potential therapeutic candidate in several neuropsychiatric disorders, especially in treating depression and anxiety [2,3]. However, the acute effects of psilocybin are reflected in altered states of consciousness (characterized by changes in perception (e.g., illusions or pseudo-hallucinations)) and altered sense of self, thinking and emotion that depend on the dosage and several individual nonpharmacological variables [4] that are not yet fully understood. Importantly, effects of psilocybin on cognitive functions have received increased attention in recent years [5,6,7], and a dose-dependent attenuation of behavioral measures related to associative learning, working and episodic memory was shown [8]. Nonetheless, the extent of the effects of psilocybin on the neural mechanisms of cognitive functioning is not yet clear.

Psilocybin has been shown to negatively affect the electrophysiological markers of both early visual (P1 and N170)/auditory (N100) [9,10,11,12] and higher-order attentive cognitive processing (P300) [9,13], but the mismatch negativity (MMN), as a marker of pre-attentive cognition [9,12], was not attenuated. Similarly, serotoninergic psychedelics, including psilocin, the active metabolite of psilocybin, have been shown recently to decrease gamma oscillations in the animal model [14]. Although gamma activity is one of the most valuable avenues for understanding the neurobiology of cognitive processing [15], the resting state gamma oscillations are difficult to study using EEG in humans due to contamination with muscle artefacts.

The 40 Hz auditory steady-state response (40 Hz ASSR) emerged as one of the biomarkers that, in a controlled manner, allows evaluation of the ability of the brain to generate gamma-range activity. Indeed, ASSRs are consistently diminished in magnitude and phase-locking level in states with disrupted cognition, especially across schizophrenic and bipolar spectrum disorders with psychosis status, and the severity of illness is linked to the extent of the reduction [16,17,18,19,20]. Furthermore, alterations of the response are seen in clinically high-risk subjects for psychosis [21,22], and a recent systematic review confirmed that gamma-range ASSRs in patients are related to executive and memory functions [23]. In studies with NMDA receptor antagonist ketamine, a dissociative anesthetic with psychedelic properties [24], 40 Hz ASSRs were shown to stand as a possible biomarker of cortical NMDA function that is translatable to schizophrenia and bipolar disorder [25,26]. Surprisingly, no prior research has evaluated the role of serotoninergic system on ASSRs in detail, although the importance of serotonin system in neurobiology of psychosis [27,28,29] and cognitive processing [30] is well known.

In order to understand whether psilocybin-induced cognitive deficits are attributable to the attenuated processing within gamma range activity, we studied the effects of acute psilocybin intoxication on ASSR in healthy volunteers. Based on the similarities of the psychedelic states induced by psilocybin or ketamine and the psychotic-like state, we hypothesized that psilocybin would, in a similar manner, impact upon ASSR measures. Specifically, we expected to observe decreased phase-locking and amplitude of 40 Hz ASSR and the individual intensity of disruption to be positively linked with the individual intensity of the psychedelic state and serum psilocin levels.

## 2. Materials and Methods

### 2.1. Participants

Participants were recruited from November 2017 to July 2018 through the snowball method and were initially pre-screened by phone interview for major inclusion/exclusion criteria (see online Supplementary Material for more detail) and if eligible, they were invited for a face-to-face interview with study investigators. After detailed introduction to the study design, effects of psilocybin, safety issues, and after answering all the participant’s questions related to the study, informed consent was obtained and, subsequently, subjects were screened by the Minnesota Multiphasic Personality Inventory (MMPI-2) [31] and Mini-International Neuropsychiatric Interview (MINI) [32] for any significant psychopathology. Participants were excluded if they screened positive for any psychiatric disorder (according to ICD-10), as well as any family history of psychotic disorder (up to second degree relatives). Participants with major physical disorders (intracranial hypertension, arterial or pulmonary hypertension, a cerebral stroke in the past, cardiac insufficiency, coeliac disease, and liver dysfunction), regular use of medication (except contraceptives), pregnancy, presence of ferromagnetic materials in their body and cardio-stimulator, and left-handedness (evaluated using the Edinburgh Handedness Inventory) were also excluded from the study.

Finally, 20 healthy volunteers were enrolled (10M/10F, mean age M = 36; SD = 8). During the screening visit, subjects were physically examined, vital sign measurements (blood pressure (BP) and heart rate (HR)) were documented, and blood samples were taken to assess liver function (plasma levels of bilirubin, alanine aminotransferase, aspartate aminotransferase and gamma-glutamyl transferase). All participants underwent a urine drug screening test. The same research team that led them through all of the subsequent measurements examined participants. The study team consisted of three people: (1) a study clinician (psychiatrist), (2) a second sitter (psychologist or psychiatrist) and (3) a laboratory EEG technician/nurse. The pair of the study clinician and the second sitter was always gender balanced. Participants were asked: (1) to remain drug-free until the day of the experiment (urine drug screening was performed on the day of testing), (2) to abstain from alcohol for at least one week prior to the session (an alcohol breath test was obtained on the day of testing), (3) not to eat anything or drink coffee on the day of the experiment, and (4) not to smoke tobacco for at least two hours prior to administration of the study medication. The study design was elaborated to correspond to the Guidelines for Safety in Human Hallucinogen Research [33].

### 2.2. Study Approval

The study was approved by the local ethical committee of National Institute of Mental Health and by Czech State Institute for Drug Control. It was approved as a clinical trial registered under the EudraCT No. 2012-004579-37.

### 2.3. Experimental Design

A study was planned as a crossover, double blind, placebo-controlled design. Each participant underwent two sessions, with the interval between measurements set for at least 28 days. On the dosing (experimental) day, participants were physically re-examined by the study clinician, vital sign measurements (BP, HR) were collected and a short, structured interview was conducted in order to (1) exclude any new possible contraindications that would make participants ineligible for the study and (2) again, shortly describe the nature of effects of psilocybin, risks, side effects and description of the measures that would be collected during the session. Participants subsequently underwent insertion of an intravenous cannula for blood sampling, and a high-density (256 channels) gel EEG net was mounted on volunteer’s head. The experiment was performed in a sound-attenuated and electrically shielded experimental room (Faraday cage) that was decorated with colored blankets on the wall, candles, and other decorative items, in order to induce a pleasant and relaxed environment. The whole session lasted approximately 6 h from drug administration. During the whole experiment, blood samples (for serum psilocin levels) and vital signs (BP/HR) were collected, as shown in the timeline in Figure S1 in the online Supplementary Material. Resting-state EEG and other ERPs were also collected but are not reported here.

### 2.4. Psilocybin Dosage

Psilocybin was manufactured according to good manufacturing practice standards from THC-Pharm GmbH, Frankfurt, Germany. Gelatin capsules containing 1 and 5 mg of psilocybin homogenized with Trittici amylum were prepared in the pharmacy of the Institute for Clinical and Experimental Medicine in Prague, Czech Republic. The dosage was set according to the weight of the participant to be approximately 0.26 mg/kg, which should induce psychotic-like symptoms [34,35]. The dose was increased by 1 mg per 5 kg of body weight. The drug was administered orally in an adjusted number of capsules and swallowed after drinking 200 mL of water.

### 2.5. Psychological and Physiological Measures

The Brief Psychiatric Rating Scale (BPRS) [36] was administered 40 min before ingestion of the drug and again 60, 175, and 360 min after ingestion. The scale contains 18 items, rated by the researcher on a six-point, Likert-type scale ranging from “not present” to “severe/very strong”.

The Hallucinogen Rating Scale (HRS) [37], which consists of 71 items evaluating six domain scales (Somasthesia, Affect, Volition, Cognition, Perception and Intensity) was used. Participants rated each item on a four-point Likert-type scale ranging from “not at all” to “extremely”. HRS measures the perceptual, somatic and psychological effects of hallucinogenic drugs.

The Altered States of Consciousness Rating Scale (ASC) [38] was administered just after the end of session, when the symptoms of intoxication had worn off. It reflects a subjective rating of the whole experience and consists of 94 items, which are divided into 11 factors: Experience of Unity (EOU), Spiritual Experience (SE), Blissful State (BS), Insightfulness (IF), Disembodiment (DB), Impaired Control and Cognition (ICC), Anxiety (AX), Complex Imagery (CI), Elementary Imagery (EI), Audio-Visual Synaesthesia (AVS), and Changed Meaning of Percepts (CMP). ASC measures deviation in the subjective experience or psychological functioning of a normal individual from her/his usual waking consciousness [39].

### 2.6. Auditory Stimulation

The auditory steady-state response was recorded 10 min before the ingestion of the drug and again ~105 min after drug ingestion, around the typical peak of experience and when most pronounced psychotic-like symptoms were expected [34,40]. Subjects were laying in the bed with their eyes closed and instructed to focus on the stimulation.

The 40 Hz click stimulation trials lasted 500 ms and consisted of 20 identical clicks. Each 40 Hz trial was presented 150 times, with an inter-stimulus interval set at 700–1000 ms. Sounds were presented binaurally through Sennheiser HD 280 earphones; the sound pressure level was adjusted to 60 dB with an AZ8922 digital sound level meter (AZ Instrument Corp., Taichung City, Taiwan). The click train onset was corrected for jitter and tested by the EGI AV TESTER hardware.

### 2.7. EEG Recording

Data were recorded using EGI 256-channel EEG system equipped by Net Amps 400 series amplifier, Fs = 1000 Hz, DC coupling with 256 HydroCel Geodesic Sensor Net 220 MR, and Net Station 5 acquisition software.

### 2.8. Data Analysis

#### 2.8.1. EEG Data Pre-Processing

The off-line pre-processing of the EEG data was performed with a BrainVision Analyzer v. 2.2 (BVA). Firstly, the data were filtered by an IIR filter (range 1–200 Hz) and a 50 Hz notch was used. Then, the data were screened visually for any major artefacts caused by muscle activity, and artefactual segments (e.g., those generated by rough head movements etc.) were removed from further processing. As, especially during the psilocybin intoxication, participants were clenching their jaws, the outer range electrodes were rejected from further evaluation. Among the remaining 173 electrodes (layout plotted in Figure S2, Supplementary Material), channels with excessive noise/artefacts were determined by careful visual inspection and removed, in order not to contaminate the subsequent independent component analysis (ICA). Removed channels were replaced using spherical spline interpolation of the voltage from surrounding electrodes [41]. As 40 Hz ASSRs are sensitive to fluctuations in arousal level [42,43], the data were carefully visually screened to exclude drowsiness and sleep periods: segments with continuous NREM I states lasting more than 15 sec were excluded, based on standard criteria [44]. Subsequently, in order to exclude eye blinks and eye movements, ICA built-in BVA, followed by the manual elimination of corresponding components (based on typical graphoelements and topographic maps) were performed, and built-in inverse ICA was used to recompose the data. Afterwards, the signal was re-referenced to the average of the electrodes. The epochs of 1100 ms were selected within the time windows, starting at 250 ms prior to the stimulus onset and lasting for 850 ms post-stimulus onset.

A wavelet transformation and further time-frequency analyses were performed in MATLAB and ERPWAVELAB [45]. The complex Morlet wavelet from MATLAB© Wavelet Toolbox, with frequencies represented from 1 to 60 Hz in 1 Hz intervals between each frequency, was used for wavelet transformation. The phase-locking index (PLI), time-frequency transformed evoked potential (EA), and event-related spectral perturbation (ERSP) measures were calculated. The PLI corresponds to the phase consistency of the response over epochs and ranges between 0 and 1. The EA corresponds to wavelet-transformed evoked potential and represents a phase-aligned amplitude measure [45]. The ERSP measure reflects mean event-related changes in amplitude of the frequency spectrum, induced in this case by auditory stimulation [46]. The PLI, EA and ERSP curves were extracted by averaging gamma activity within the 35 to 45 Hz frequency range. To focus on the late-latency gamma response [47,48,49], the signal was averaged within the 200 to 500 ms range. PLIs, EAs and ERSPs were baseline-corrected by subtracting mean activity of the pre-stimulus period (starting −150 ms before the stimulus onset until −50 ms). The ASSRs were analyzed as the average of the fronto-central electrodes (see Supplementary Material), where ASSRs are maximal.

#### 2.8.2. Statistical Evaluation

Repeated measures ANOVAs were performed to encounter the effects of the drug (psilocybin vs. placebo) and the treatment order (pre-drug vs. post-drug) as individual factors, and their potential interactions, separately, for PLI, EA and ERSP indices. In order to assess the drug’s effects in detail, planed contrasts for interaction (type: repeated) with the weights set at 0 for pre-drug, 1 for post-placebo and −1 for post-psilocybin were used, as implemented in JASP 0.14.1 [50].

To see the connection between psychological and psychiatric measures of intoxication intensity and ASSR, the scores on 11 factors of ASC, 6 factors of HRS, and the BPRS values obtained at 60 and 175 min were correlated to PLI, EA and ERSP measures obtained after psilocybin. Additionally, the intensity of the effect measured as the pre-psilocybin to post-psilocybin difference for PLI, EA and ERSP values were correlated to the scales. Pearson correlation coefficients and other statistics that are part of Supplementary Material were calculated using IBM SPSS Statistics 22.

## 3. Results

Out of 20 enrolled subjects, the final sample consisted of 12 subjects. The data of eight subjects could not be used due to the following reasons: two subjects had decreased vigilance during the placebo session, one was excluded due to premature termination (subject participated in one session only), and five other subjects were excluded due to insufficient data quality. Several participants had a previous history of psychedelic use (*n* = 6) and others were drug-naive (*n* = 6). Five participants had previous experience with psilocybin (not fulfilling F16 diagnosis).

### 3.1. Psilocin Pharmacokinetics

The average dose of psilocybin used was 17.83 mg (15–21 mg). The highest psilocin serum levels, 13.63 ng/mL (SD ± 4.61), were observed at 120 min after ingestion, then dropped to 7.46 ng/mL (SD ± 2.02) at 240 min and to less than 5 ng/mL 360 min after ingestion (Table S1, online Supplemental Material).

### 3.2. Effects of Psilocybin on Subjective Experience and Psychopathology

**Figure 1:**
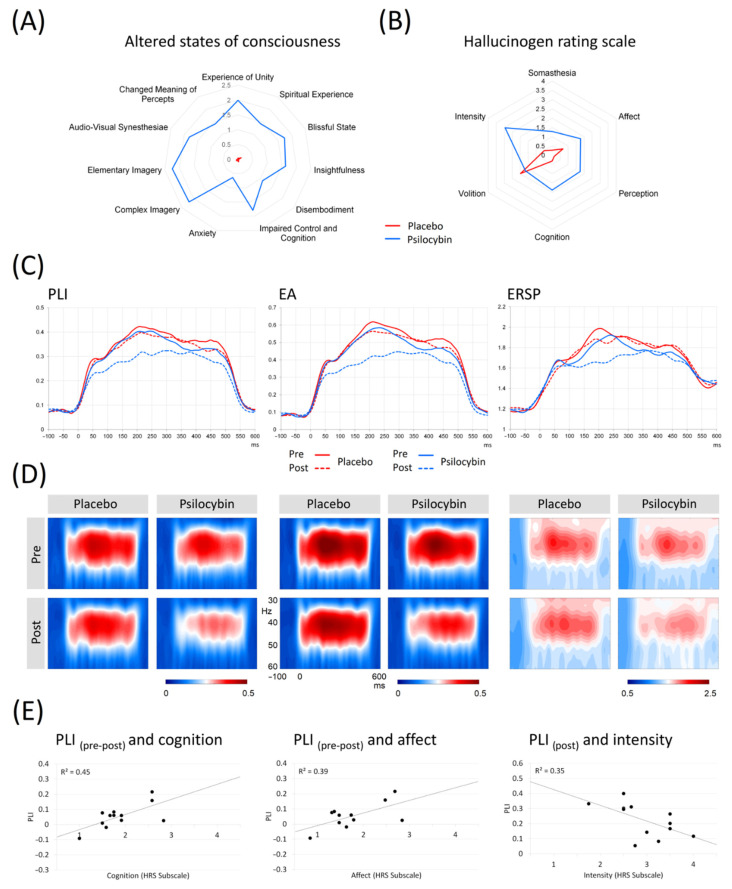
(**A**) The graph shows comparisons of 11 factors from the Altered states of consciousness scale, including the clustering of factors into three main subscales Oceanic boundlessness, Self-disintegration and Perception in 12 subjects during psilocybin intoxication vs. placebo. (**B**) The graph shows comparison of 6 factors from the Hallucinogen rating scale in 12 subjects during psilocybin intoxication vs. placebo. (**C**) Grand averages of the PLI, EA and ERSP curves across fronto-central ROI in pre-placebo, post-placebo, pre-psilocybin, and post-psilocybin conditions. (**D**) Time-frequency plots of PLI, EA and ERSP. (**E**) Scatterplots showing significant correlations between PLI difference values and facets of HRS—affect, cognition; scatterplot showing significant correlation between PLI values in the peak of intoxication and HRS facet intensity. Legend: PLI = Phase-locking index; ERSP = Event-related spectral perturbation; EA = Evoked amplitude; Pre = measurement before drug intake; Post = measurement in the peak of intoxication.

Analyses of ASCs revealed a significant effect of psilocybin compared to the placebo in all subscales. BPRS measured at 60 and 175 min also revealed a significant effect and, similarly, the effects of the treatment were significant in almost all subscales of HRS (see Figure 1A,B and the online Supplementary Material for more information).

### 3.3. Vital Signs

At the peak of intoxication (60 min after ingestion), psilocybin led to a mild but significant increase in systolic and diastolic BP of 13–21 mmHg, but not an increase in heart rate (for details see online Supplementary Material).

### 3.4. 40 Hz Auditory Steady-State Response

**Figure 2:**
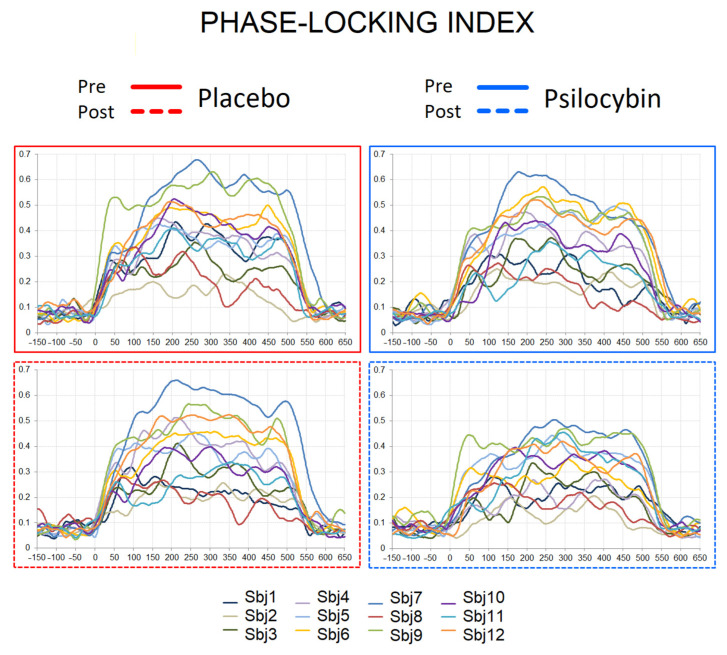
Individual PLI curves in pre-placebo, post-placebo, pre-psilocybin and post-psilocybin conditions. X axis—time in milliseconds, Y axis—PLI values.

The statistical evaluation was performed on the fronto-central ROI, where 40 Hz ASSRs show maximal activation (see Figure S2 in Supplementary Material for electrodes) [51,52]. The grand-averaged PLI, EA and ERSP curves for pre/post placebo and pre/post psilocybin conditions are plotted in Figure 1D. The individual PLI curves in all experimental conditions are presented in Figure 2. Means and standard deviations of PLI, EA and ERSP before and after placebo and before and after psilocybin are presented in Table S2 in the online Supplementary Material.

#### 3.4.1. Phase-Locking Index (PLI)

Significant effects of drug (psilocybin vs. placebo), F (1,11) = 7.301, *p* = 0.021, η_p_^2^ = 0.399 and of treatment order (pre-drug vs. post-drug), F (1,11) = 10.097, *p* = 0.009, η_p_^2^ = 0.479 were observed. The interaction between the factors was not significant, F (1,11) = 1.081, *p* = 0.321, η_p_^2^ = 0.089; however, the planned contrast analysis for interaction was significant (t = 2.532, *p* = 0.019) for the weights 0 0 1 −1, indicating that PLIs at the pre-placebo and pre-psilocybin stages did not differ, and post-placebo PLIs were higher than post-psilocybin values.

#### 3.4.2. Evoked Amplitude (EA)

We found significant effects for drug condition, F (1,11) = 7.842, *p* = 0.017, η_p_^2^ = 0.416 and treatment order, F (1,11) = 11.857, *p* = 0.005, η_p_^2^ = 0.519, but no interaction between the drug and treatment order was observed (F (1,11) = 1.263, *p* = 0.285, η_p_^2^ = 0.103). However, planned contrast analysis for the interaction was significant (t = 2.721, *p* = 0.013) for the weights 0 0 1 −1, indicating that EAs at the pre-placebo and pre-psilocybin stages were similar, and post-placebo EAs were higher than post-psilocybin measures.

#### 3.4.3. Event-Related Spectral Perturbation (ERSP)

We did not find significant effects of drug condition, F (1,11) = 4.764, *p* = 0.052, η_p_^2^ = 0.302, but significant effects of treatment order, F (1,11) = 6.282, *p* = 0.029, η_p_^2^ = 0.363 were observed. No interaction between drug and treatment order was observed, F (1,11) = 0.358, *p* = 0.562, η_p_^2^ = 0.032. A planned contrast analysis for interaction was not significant (t = 2.002, *p* = 0.058) for the weights 0 0 1 −1, indicating that ERSPs at the pre-placebo and pre-psilocybin stages did not differ, and post-placebo ERSPs tended to be higher than values at post-psilocybin.

### 3.5. ASSR Correlations with Psychometric Measures and Psilocin Plasma Levels

Only the scores on the Intensity subscale of HRS were negatively correlated to PLI values obtained after psilocybin intoxication (r = −0.59, *p* < 0.05). A significant relationship between the intensity effect for PLI and the two subscales of HRS was observed: Cognition (r = 0.674, *p* < 0.05) and Affect (r = 0.628, *p* < 0.05) (see Table S3 in online Supplementary Material). A positive relationship between Cognition and intensity effect for EA (r = 0.605, *p* < 0.05) was also evident. There were no other significant correlations between the 11 factors of the ASC and any of the measures of ASSR (PLI, ERSP and EA, see Table S5 in online Supplementary Material). There was no significant relationship between BPRS measured at 60 min and 175 min and any of the ASSR measures (see Table S6 in online Supplementary Material). There was no significant relationship between the measures of ASSR and the level of psilocin in the blood samples at 120 min. The results are shown in Table S7 in the online Supplementary Material.

## 4. Discussion

The main finding of our study is that psilocybin intoxication resulted in a significant reduction of phase-locked measures of 40 Hz ASSRs. Moreover, the intensity of the effect (pre-post difference) for the phase-locking was strongly related to the state of Cognition and Affect according to the HRS scales, and the phase-locking after psilocybin intoxication was inversely related to the Intensity scores of HRS. Importantly, despite the fact that the reduced PLI and EA were observed during the peak of psilocybin intoxication, we did not find any correlations with the plasma levels of psilocin at 120 min after ingestion.

Psilocybin, acting as an agonist at serotonin 5-HT2A/C and 5-HT1A receptors [1] is known to mimic positive-like symptoms and, thus, it is also used as a serotoninergic model of psychosis [27,40]. Indeed, a single dose of psilocybin produced a significant increase in psychotic symptoms (Supplementary Material); however, no correlations between ASSR measures and the measures evaluated by the ASCS and BPRS scales were observed. The 40 Hz ASSRs were suggested as potential biomarkers of psychosis [53] and sensitive marker of excitation/inhibition (E/I) balance in the brain [49,54], as supported by numerous animal studies targeting glutamate- and GABA-ergic transmission [26,55,56]. Previous studies in animal models demonstrated that 5-HT2A and 5-HT1A receptors finely tune the amplitude of gamma oscillations [57], and serotonin-boosting medications suppress gamma activity [58,59], possibly through 5-HT1AR [57]. Our recent study [14] showed the global desynchronization of gamma activity by tryptamines (such as psilocin) administered to rats. Thus, we expected that psilocybin would attenuate the gamma response in a similar manner, as seen in various states with disrupted E/I balance and cognition, including psychosis or during intoxication with other psychedelic substances, such as ketamine [25,26,54]. The current findings on the phase-locked measures of 40 Hz ASSRs fully support this notion, and the insignificant finding for the ERSP that reflects the event-related changes in power relative to a pre-stimulus baseline may probably be attributed to the higher susceptibility of this measure to noise in EEG signal, compared to PLI and EA. Moreover, the results, alongside the previous reports on the reduced phase synchronization and power of 40 Hz ASSRs in cannabinoid models of psychosis [60] and increased power in dopaminergic model [61], suggest a possible link between 5-HT, cannabinoid, dopaminergic and, especially, NMDA functioning. However, more research is needed to elucidate this connection with respect to the characteristic psychological effect shared by all these pharmacological manipulations.

It was shown earlier that psilocybin reduces coupling between the posterior cingulate cortex and the medial prefrontal cortex [62]; these areas were recently shown to contribute to 40 Hz ASSRs [63], and are implicated in memory and executive control [64]. Overall, the observation of reduced 40 Hz ASSRs after psilocybin ingestion is in congruence with the assumption that, under the influence of serotoninergic psychedelics, the “primary states” of the brain are elevated, so the brain function is disorganized, affecting reality-testing and self-awareness [65]. Importantly, the reduction of ASSRs during psilocybin intoxication was evident only in subjects with initially stronger ASSRs (details in Figure 2). This suggests that at a certain low level of inhibition/excitation balance, as indexed by ASSR, the drug’s effects are not prominent. This observation is in accordance with the recent report [66] in patients with schizophrenia, where subjects with larger ASSR responses had more robust cognitive gains in response to targeted cognitive training. It is possible that the “baseline” (i.e., pre-intervention) gamma activity may stand as an index of the brain’s overall “adaptive integrity“ of its lower-level perceptual networks. With this line of thinking in mind, the positive relationship between the intensity of PLI change and Cognition and Affect subscales of HRS (meaning stronger distortion of cognition and affect alongside the larger drug-induced reduction in PLIs) is an interesting finding, as, in our previous work, we have seen similar linkage between these measures and P300 [9]. This is also supported by a negative correlation between the Intensity scores of HRS (measuring the state of the drug effect) and PLI values after the psilocybin intoxication. It is plausible that experiencing intense emotional states contributes to the disturbance of cognitive processing [67], as serotonin possibly relaxes prior assumptions to habitual responses and 5-HT2AR mediates enhanced brain plasticity [68]. Under serotoninergic psychedelics, the brain approaches criticality [69], and is thus sensitive to perturbation, while phase synchronization of the brain and, possibly, executive functioning are attenuated. Taken together, our findings yield further support for the role of gamma oscillations in cognitive processing and its disturbance.

Finally, the complex methodology of the current study, including collection of the ASSRs in each participant four times, allowed an evaluation of the test-retest stability of the response. Several previous works reported a good test-retest stability of 40 Hz ASSRs in both clinical [70,71] and healthy samples [72,73]. Our observation goes further by providing evidence that ASSRs remain individually consistent, even after pharmacological intervention (see Tables S8–S10 in the online Supplementary Material).

### Limitations

The main limitation of this study is a relatively small final sample size. Future studies should seek to enroll larger groups of participants, and potentially not exclude data based on the signs of early sleep stages. Nevertheless, the double blind, placebo-controlled investigation with a crossover design allowed evaluation of the effect of interest.

## 5. Conclusions

The current study showed that psilocybin lowered the synchronization level and the amplitude of 40 Hz auditory steady-state responses. These changes were associated with subjective experiences of Affect, Cognition and Intensity. The result yields further support for the role of gamma oscillations in cognitive processing and its disturbance.

## Data Availability

The data presented in this study are available on request from the corresponding author. The data are not publicly available due to privacy restrictions.

## Supplementary Materials

The following supporting information can be downloaded at: https://www.mdpi.com/article/10.3390/jpm12061004/s1, Figure S1: The timeline of the experimental session; Figure S2: 256 channel EEG map; Figure S3: Mean difference in blood pressure and heart rate during psilocybin intoxication; Figure S4: Mean values for Factors 1–5 of BPRS for psilocybin intoxication during baseline, at 60 and 180 min and end of session 360 min; Table S1: Psilocin plasma levels during intoxication with psilocybin; Table S2: Means and standard deviations of three ASSR measures; Table S3: Pearson correlation coefficient of HRS questionnaire and difference measures of ASSR; Table S4: Pearson correlation coefficient of HRS questionnaire and measures of ASSR in the peak of intoxication.; Table S5: Pearson correlation coefficient of 5D-ASC questionnaire and measures of ASSR; Table S6: Pearson correlation coefficient of BPRS measured in 60 and 175 min since administration of psilocybin and measures of ASSR; Table S7: Pearson correlation coefficient of psilocin level in 120 min and measures of ASSR measured in the peak of intoxication; Table S8: Pearson correlation coefficient of measure of ASSR—Evoked amplitude; Table S9: Pearson correlation coefficient of measure of ASSR—Phase-locking index; Table S10: Pearson correlation coefficient of measure of ASSR—Event-related spectral perturbation. Refs. [9,74,75] cited in Supplementary.

## References

[1] Nutt D. (2019). Psychedelic drugs—A new era in psychiatry?. Dialog-Clin. Neurosci..

[2] Li N.-X., Hu Y.-R., Chen W.-N., Zhang B. (2021). Dose effect of psilocybin on primary and secondary depression: A preliminary systematic review and meta-analysis. J. Affect. Disord..

[3] Więckiewicz G., Stokłosa I., Piegza M., Gorczyca P., Pudlo R. (2021). Lysergic Acid Diethylamide, Psilocybin and Dimethyltryptamine in Depression Treatment: A Systematic Review. Pharmaceuticals.

[4] Studerus E., Gamma A., Kometer M., Vollenweider F.X. (2012). Prediction of Psilocybin Response in Healthy Volunteers. PLoS ONE.

[5] Meinhardt M.W., Pfarr S., Fouquet G., Rohleder C., Meinhardt M.L., Barroso-Flores J., Hoffmann R., Jeanblanc J., Paul E., Wagner K. (2021). Psilocybin targets a common molecular mechanism for cognitive impairment and increased craving in alcoholism. Sci. Adv..

[6] Vollenweider F.X., Preller K.H. (2020). Psychedelic drugs: Neurobiology and potential for treatment of psychiatric disorders. Nat. Rev. Neurosci..

[7] Doss M.K., Považan M., Rosenberg M.D., Sepeda N.D., Davis A.K., Finan P.H., Smith G.S., Pekar J.J., Barker P.B., Griffiths R.R. (2021). Psilocybin therapy increases cognitive and neural flexibility in patients with major depressive disorder. Transl. Psychiatry.

[8] Barrett F.S., Carbonaro T.M., Hurwitz E., Johnson M.W., Griffiths R.R. (2018). Double-blind comparison of the two hallucinogens psilocybin and dextromethorphan: Effects on cognition. Psychopharmacologia..

[9] Bravermanová A., Viktorinová M., Tylš F., Novák T., Androvičová R., Korčák J., Horáček J., Balíková M., Griškova-Bulanova I., Danielová D. (2018). Psilocybin disrupts sensory and higher order cognitive processing but not pre-attentive cognitive processing—study on P300 and mismatch negativity in healthy volunteers. Psychopharmacology.

[10] Kometer M., Cahn B.R., Andel D., Carter O.L., Vollenweider F.X. (2011). The 5-HT2A/1A Agonist Psilocybin Disrupts Modal Object Completion Associated with Visual Hallucinations. Biol. Psychiatry.

[11] Kometer M., Schmidt A., Jäncke L., Vollenweider F.X. (2013). Activation of Serotonin 2A Receptors Underlies the Psilocybin-Induced Effects on α Oscillations, N170 Visual-Evoked Potentials, and Visual Hallucinations. J. Neurosci..

[12] Umbricht D.S., Vollenweider F.X., Schmid L., Grübel C., Skrabo A., Huber T., Koller R. (2003). Effects of the 5-HT2A Agonist Psilocybin on Mismatch Negativity Generation and AX-Continuous Performance Task: Implications for the Neuropharmacology of Cognitive Deficits in Schizophrenia. Neuropsychopharmacology.

[13] Kometer M., Schmidt A., Bachmann R., Studerus E., Seifritz E., Vollenweider F.X. (2012). Psilocybin biases facial recognition, goal-directed behavior, and mood state toward positive relative to negative emotions through different serotonergic subreceptors. Biol Psychiatry.

[14] Vejmola Č., Tylš F., Piorecká V., Koudelka V., Kadeřábek L., Novák T., Páleníček T. (2021). Psilocin, LSD, mescaline, and DOB all induce broadband desynchronization of EEG and disconnection in rats with robust translational validity. Transl. Psychiatry.

[15] Başar E. (2013). A review of gamma oscillations in healthy subjects and in cognitive impairment. Int. J. Psychophysiol..

[16] Isomura S., Onitsuka T., Tsuchimoto R., Nakamura I., Hirano S., Oda Y., Oribe N., Hirano Y., Ueno T., Kanba S. (2016). Differentiation between major depressive disorder and bipolar disorder by auditory steady-state responses. J. Affect. Disord..

[17] Light G.A., Hsu J.L., Hsieh M.H., Meyer-Gomes K., Sprock J., Swerdlow N.R., Braff D.L. (2006). Gamma Band Oscillations Reveal Neural Network Cortical Coherence Dysfunction in Schizophrenia Patients. Biol. Psychiatry.

[18] Parker D.A., Hamm J.P., McDowell J.E., Keedy S.K., Gershon E.S., Ivleva E.I., Pearlson G.D., Keshavan M.S., Tamminga C.A., Sweeney J.A. (2019). Auditory steady-state EEG response across the schizo-bipolar spectrum. Schizophr. Res..

[19] Spencer K.M., Salisbury D.F., Shenton M.E., McCarley R.W. (2008). γ-Band Auditory Steady-State Responses Are Impaired in First Episode Psychosis. Biol. Psychiatry.

[20] Thuné H., Recasens M., Uhlhaas P.J. (2016). The 40-Hz Auditory Steady-State Response in Patients With Schizophrenia. JAMA Psychiatry.

[21] Ahmed S., Lepock J.R., Mizrahi R., Bagby R.M., Gerritsen C.J., Korostil M., Light G.A., Kiang M. (2020). Decreased Gamma Auditory Steady-State Response Is Associated With Impaired Real-World Functioning in Unmedicated Patients at Clinical High Risk for Psychosis. Clin. EEG Neurosci..

[22] Tada M., Nagai T., Kirihara K., Koike S., Suga M., Araki T., Kobayashi T., Kasai K. (2016). Differential Alterations of Auditory Gamma Oscillatory Responses between Pre-Onset High-Risk Individuals and First-Episode Schizophrenia. Cereb. Cortex.

[23] Parciauskaite V., Bjekic J., Griskova-Bulanova I. (2021). Gamma-Range Auditory Steady-State Responses and Cognitive Performance: A Systematic Review. Brain Sci..

[24] Gao M., Rejaei D., Liu H. (2016). Ketamine use in current clinical practice. Acta Pharmacol. Sin..

[25] Plourde G., Baribeau J., Bonhomme V. (1997). Ketamine increases the amplitude of the 40-Hz auditory steady-state response in humans. Br. J. Anaesth..

[26] Sivarao D.V., Chen P., Senapati A., Yang Y., Fernandes A., Benitex Y., Whiterock V., Li Y.-W., Ahlijanian M.K. (2016). 40 Hz Auditory Steady-State Response Is a Pharmacodynamic Biomarker for Cortical NMDA Receptors. Neuropsychopharmacology.

[27] Geyer M.A., Vollenweider F.X. (2008). Serotonin research: Contributions to understanding psychoses. Trends Pharmacol. Sci..

[28] Kantrowitz J.T. (2020). Targeting Serotonin 5-HT2A Receptors to Better Treat Schizophrenia: Rationale and Current Approaches. CNS Drugs.

[29] Stahl S.M. (2018). Beyond the dopamine hypothesis of schizophrenia to three neural networks of psychosis: Dopamine, serotonin, and glutamate. CNS Spectr..

[30] Švob Štrac D., Pivac N., Mück-Šeler D. (2016). The serotonergic system and cognitive function. Transl. Neurosci..

[31] Butcher J.N., Graham J.R., Fowler R.D. (1991). Special Series: The Mmpi-2*. J. Pers. Assess..

[32] Sheehan D.V., Lecrubier Y., Sheehan K.H., Amorim P., Janavs J., Weiller E., Hergueta T., Baker R., Dunbar G.C. (1998). The Mini-International Neuropsychiatric Interview (M.I.N.I): The development and validation of a structured diagnostic psychiatric interview for DSM-IV and ICD-10. J. Clin. Psychiatry.

[33] Johnson M., Richards W., Griffiths R. (2008). Human hallucinogen research: Guidelines for safety. J. Psychopharmacol..

[34] Nichols D.E. (2016). Psychedelics. Pharmacol. Rev..

[35] Tylš F., Páleníček T., Kaderábek L., Lipski M., Kubešová A., Horácek J. (2016). Sex differences and serotonergic mechanisms in the behavioural effects of psilocin. Behav. Pharmacol..

[36] Overall J.E., Hollister L.E., Pichot P. (1967). Major Psychiatric Disorders: A Four-Dimensional Model. Arch. Gen. Psychiatry.

[37] Strassman R.J., Qualls C.R., Uhlenhuth E.H., Kellner R. (1994). Dose-Response Study of N,N-Dimethyltryptamine in Humans: II. Subjective Effects and Preliminary Results of a New Rating Scale. Arch. Gen. Psychiatry.

[38] Studerus E., Gamma A., Vollenweider F.X. (2010). Psychometric Evaluation of the Altered States of Consciousness Rating Scale (OAV). PLoS ONE.

[39] Dittrich A. (1998). The standardized psychometric assessment of altered states of consciousness (ASCs) in humans. Pharmacopsychiatry.

[40] Tylš F., Páleníček T., Horáček J. (2014). Psilocybin–Summary of knowledge and new perspectives. Eur. Neuropsychopharmacol..

[41] Perrin F., Pernier J., Bertrand O., Echallier J. (1989). Spherical splines for scalp potential and current density mapping. Electroencephalogr. Clin. Neurophysiol..

[42] Górska U., Binder M. (2019). Low and medium frequency auditory steady-state responses decrease during NREM sleep. Int. J. Psychophysiol..

[43] Griskova I., Morup M., Parnas J., Ruksenas O., Arnfred S.M. (2007). The amplitude and phase precision of 40 Hz auditory steady-state response depend on the level of arousal. Exp. Brain Res..

[44] Iber C., Ancoli-Israel S., Chesson A., Quan S. (2007). The AASM Manual for the Scoring of Sleep and Associated Events: Rules, Terminology and Technical Specifications. Am. Acad. Sleep Med..

[45] Mørup M., Hansen L.K., Arnfred S.M. (2007). ERPWAVELAB: A toolbox for multi-channel analysis of time–frequency transformed event related potentials. J. Neurosci. Methods.

[46] Makeig S. (1993). Auditory event-related dynamics of the EEG spectrum and effects of exposure to tones. Electroencephalogr. Clin. Neurophysiol..

[47] Griskova-Bulanova I., Hubl D., van Swam C., Dierks T., Koenig T. (2016). Early- and late-latency gamma auditory steady-state response in schizophrenia during closed eyes: Does hallucination status matter?. Clin. Neurophysiol..

[48] Griskova-Bulanova I., Dapsys K., Melynyte S., Voicikas A., Maciulis V., Andruskevicius S., Korostenskaja M. (2018). 40 Hz auditory steady-state response in schizophrenia: Sensitivity to stimulation type (clicks versus flutter amplitude-modulated tones). Neurosci. Lett..

[49] Tada M., Kirihara K., Koshiyama D., Fujioka M., Usui K., Uka T., Komatsu M., Kunii N., Araki T., Kasai K. (2019). Gamma-Band Auditory Steady-State Response as a Neurophysiological Marker for Excitation and Inhibition Balance: A Review for Understanding Schizophrenia and Other Neuropsychiatric Disorders. Clin. EEG Neurosci..

[50] Love J., Selker R., Verhagen J., Marsman M., Gronau Q.F., Jamil T., Smira M., Epskamp S., Wild A., Ly A. (2015). Software to Sharpen Your Stats. APS Obs..

[51] Parciauskaite V., Voicikas A., Jurkuvenas V., Tarailis P., Kraulaidis M., Pipinis E., Griskova-Bulanova I. (2019). 40-Hz auditory steady-state responses and the complex information processing: An exploratory study in healthy young males. PLoS ONE.

[52] Voicikas A., Niciute I., Ruksenas O., Griskova-Bulanova I. (2016). Effect of attention on 40 Hz auditory steady-state response depends on the stimulation type: Flutter amplitude modulated tones versus clicks. Neurosci. Lett..

[53] O’Donnell B.F., Vohs J.L., Krishnan G.P., Rass O., Hetrick W.P., Morzorati S.L., Başar E., Başar-Eroĝlu C., Özerdem A., Rossini P.M., Yener G.G. (2013). Chapter 6-The auditory steady-state response (ASSR): A translational biomarker for schizophrenia. Supplements to Clinical Neurophysiology.

[54] Kozono N., Honda S., Tada M., Kirihara K., Zhao Z., Jinde S., Uka T., Yamada H., Matsumoto M., Kasai K. (2019). Auditory Steady State Response; nature and utility as a translational science tool. Sci. Rep..

[55] Vohs J.L., Chambers R.A., Krishnan G.P., O’Donnell B.F., Berg S., Morzorati S.L. (2010). GABAergic modulation of the 40 Hz auditory steady-state response in a rat model of schizophrenia. Int. J. Neuropsychopharmacol..

[56] Vohs J.L., Chambers R.A., O’Donnell B.F., Krishnan G.P., Morzorati S.L. (2012). Auditory steady state responses in a schizophrenia rat model probed by excitatory/inhibitory receptor manipulation. Int. J. Psychophysiol..

[57] Puig M.V., Watakabe A., Ushimaru M., Yamamori T., Kawaguchi Y. (2010). Serotonin Modulates Fast-Spiking Interneuron and Synchronous Activity in the Rat Prefrontal Cortex through 5-HT1A and 5-HT2A Receptors. J. Neurosci..

[58] Akhmetshina D., Zakharov A., Vinokurova D., Nasretdinov A., Valeeva G., Khazipov R. (2016). The serotonin reuptake inhibitor citalopram suppresses activity in the neonatal rat barrel cortex in vivo. Brain Res. Bull..

[59] Méndez P., Pazienti A., Szabó G., Bacci A. (2012). Direct Alteration of a Specific Inhibitory Circuit of the Hippocampus by Antidepressants. J. Neurosci..

[60] Cortes-Briones J., Skosnik P.D., Mathalon D., Cahill J., Pittman B., Williams A., Sewell R.A., Ranganathan M., Roach B., Ford J. (2015). Δ9-THC Disrupts Gamma (γ)-Band Neural Oscillations in Humans. Neuropsychopharmacology.

[61] Albrecht M.A., Price G., Lee J., Iyyalol R., Martin-Iverson M.T. (2013). Dexamphetamine selectively increases 40 Hz auditory steady state response power to target and nontarget stimuli in healthy humans. J. Psychiatry Neurosci..

[62] Carhart-Harris R.L., Erritzoe D., Williams T., Stone J.M., Reed L.J., Colasanti A., Tyacke R.J., Leech R., Malizia A.L., Murphy K. (2012). Neural correlates of the psychedelic state as determined by fMRI studies with psilocybin. Proc. Natl. Acad. Sci. USA.

[63] Koshiyama D., Miyakoshi M., Joshi Y.B., Nakanishi M., Tanaka-Koshiyama K., Sprock J., Light G.A. (2021). Source decomposition of the frontocentral auditory steady-state gamma band response in schizophrenia patients and healthy subjects. Psychiatry Clin. Neurosci..

[64] Euston D.R., Gruber A.J., McNaughton B.L. (2012). The Role of Medial Prefrontal Cortex in Memory and Decision Making. Neuron.

[65] Carhart-Harris R.L. (2018). The entropic brain-revisited. Neuropharmacology.

[66] Molina J.L., Thomas M.L., Joshi Y.B., Hochberger W.C., Koshiyama D., Nungaray J.A., Cardoso L., Sprock J., Braff D.L., Swerdlow N.R. (2020). Gamma oscillations predict pro-cognitive and clinical response to auditory-based cognitive training in schizophrenia. Transl. Psychiatry.

[67] Cromheeke S., Mueller S.C. (2014). Probing emotional influences on cognitive control: An ALE meta-analysis of cognition emotion interactions. Brain Struct. Funct..

[68] Carhart-Harris R., Nutt D. (2017). Serotonin and brain function: A tale of two receptors. J. Psychopharmacol..

[69] Carhart-Harris R.L., Leech R., Hellyer P.J., Shanahan M., Feilding A., Tagliazucchi E., Chialvo D.R., Nutt D. (2014). The entropic brain: A theory of conscious states informed by neuroimaging research with psychedelic drugs. Front. Hum. Neurosci..

[70] Hirano Y., Oribe N., Onitsuka T., Kanba S., Nestor P.G., Hosokawa T., Levin M., Shenton M.E., McCarley R.W., Spencer K.M. (2020). Auditory Cortex Volume and Gamma Oscillation Abnormalities in Schizophrenia. Clin. EEG Neurosci..

[71] Roach B.J., D’Souza D.C., Ford J.M., Mathalon D.H. (2019). Test-retest reliability of time-frequency measures of auditory steady-state responses in patients with schizophrenia and healthy controls. NeuroImage Clin..

[72] McFadden K.L., Steinmetz S.E., Carroll A.M., Simon S.T., Wallace A., Rojas D.C. (2014). Test-Retest Reliability of the 40 Hz EEG Auditory Steady-State Response. PLoS ONE.

[73] Tan H.R.M., Gross J., Uhlhaas P.J. (2015). MEG—measured auditory steady-state oscillations show high test–retest reliability: A sensor and source-space analysis. NeuroImage.

[74] Kamata T., Nishikawa M., Katagi M., Tsuchihashi H. (2003). Optimized glucuronide hydrolysis for the detection of psilocin in human urine samples. J. Chromat. B..

[75] Martin R., Schürenkamp J., Pfeiffer H., Lehr M., Köhler H. (2014). Synthesis, hydrolysis and stability of psilocin glucuronide. Forens. Sci. Intern..

