# Supplementary material for: Psilocybin—Mediated Attenuation of Gamma Band Auditory Steady-State Responses (ASSR) Is Driven by the Intensity of Cognitive and Emotional Domains of Psychedelic Experience
Source: J Pers Med. 2022 Jun 19;12(6):1004. doi: 10.3390/jpm12061004 (PMC9225116; doi:10.3390/jpm12061004)
Supplement: Supplementary file 1 [file jpm-12-01004-s001.zip › jpm-1771312-supplementary.pdf]

ONLINE SUPPLEMENTAL MATERIAL

Inclusion and Exclusion criteria of participants

Inclusion Criteria:

a) Men and women ages 28 to 65
b) Healthy volunteers with no psychiatric history (mental illnesses included in ICD 10 F0.X - F99.X)
c) No family history of psychotic disorders, up to second-degree relatives

Exclusion Criteria:

a) Pregnancy
b) Intracranial hypertension, arterial hypertension or pulmonary hypertension
c) Stroke
d) Congestive heart failure
e) Pacemaker
f) Metal clamp in the head and/or face
g) Celiac disease
h) Left-handedness
i) Usage of any medication on daily basis, except for contraception
j) Person in dependent position – students of medical faculties
k) Person in dependent position – students of other faculties younger than 28

Criteria for premature termination of participation in this study:

a) Participant could cancel their participation anytime, without giving any reason
b) Changes in health state, including use of pharmacological substances
c) Changes in psychic state, including traumatic events in the last 3 months (e.g., death in family etc.)
d) Pregnancy
e) Significant side effects that are related to assessments in this study

The timeline of the experimental session

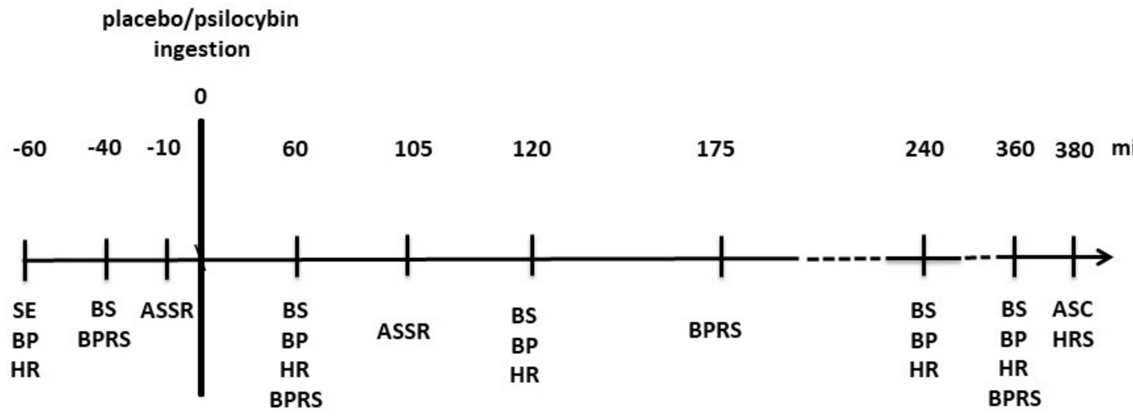

Figure S1. The timeline of the experimental session before and after ingestion of placebo/psilocybin capsules. Legend: ASC = Altered States of Consciousness Rating Scale; ASSR = auditory steady-state responses; BP= blood pressure; BPRS = Brief Psychiatric Rating Scale; BS = blood sample; HR = heart rate; HRS = Hallucinogen Rating Scale

256 channel EEG map – EGI HydroCel Geodesic Sensor Net™

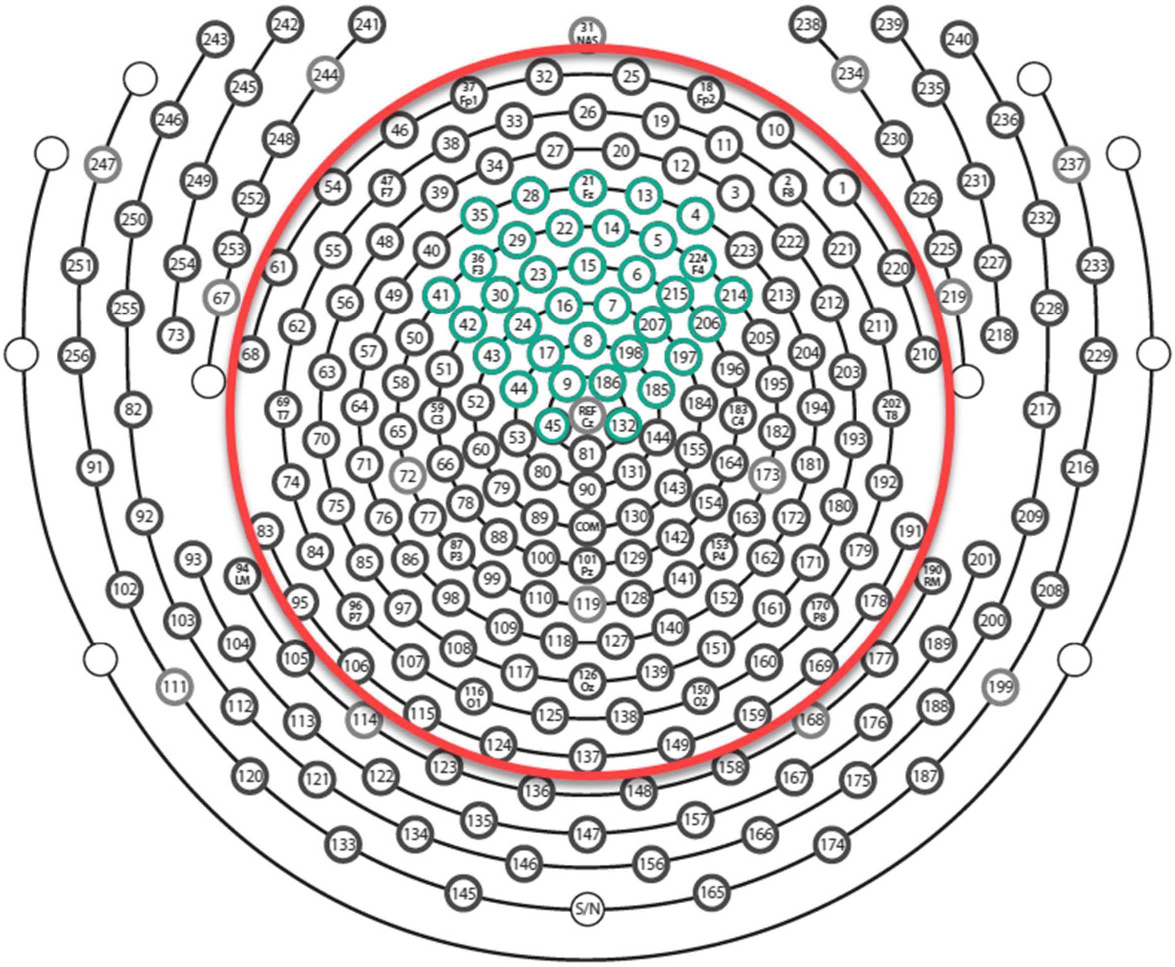

Figure S2. Illustration of 256 EEG channel map. All the electrodes (173) in the red circle were used in pre-processing of the data and analysis. The results were calculated on the fronto-central ROI (light green coloured electrodes), where 40Hz ASSR show maximal activation.

Psilocin levels

The samples were analysed after the extensive optimization and validation of the sample preparation procedure, according to the 2001 FDA Guidance using the LC-MS method. Serum sample preparation consisted of protein precipitation and was following: 1) 200 μL serum with IS was diluted with 200 μL 0.1% acetic acid and vortexed; 2) 2×400 μL acetonitrile was added to a mixture which was immediately vortexed after each contribution; 3) centrifugation for 10 min (12,000 RPM) at 5°C; 4) evaporation of 960 μL supernatant to dryness (Centrivap Concentrator); and 5) reconstitution with 10 mM formic acid in water/methanol, 95/5 (v/v). The procedure for the serum sample preparation with hydrolysis was based on studies by [1] and [2]; and was the following: 1) 10 μL 0.1 M ascorbic acid was added to 100 μL serum with IS and the mixture was vortexed; 2) 290 μl 50 mM acetic buffer (pH 4.5) with beta-glucuronidasis (500U)

was added, the content was shaken and put into a dry bath (37°C for 4 h); 3) 2×400 μL acetonitrile was added to a mixture, which was immediately vortexed after each contribution; 4) centrifugation for 10 min (12,000 RPM) at 5°C; 5) evaporation of 960 μL supernatant to dryness (Centrivap Concentrator); and 6) reconstitution with 10 mM formic acid in water/methanol, 95/5 (v/v). LC-MS analysis: all samples were analysed using UHPLC-MS/MS instrumentation (1290 Infinity Agilent Technologies Agilent 6460 Triple Quadrupole LC/MS with Agilent Jet Stream electrospray ionization source). A column Agilent Zorbax Eclipse RRHD (50 × 2.1 mm, 1.8 μm) with a pre-column was used for a chromatographic separation, with gradient elution in the system of 0.1% (v/v) formic acid (mobile phase A) and methanol (mobile phase B). Data were acquired through a positive electrospray ionization (ESI) mode by a multiple reaction monitoring method (MRM). Psilocin was quantified using an external matrix-matched calibration and deuterated internal standard psilocin-d10.

	Time				
	0 min	60 min	120 min	240 min	360 min
Sbj 1	0	9	14	8	5
Sbj 2	0	8	23	7	4
Sbj 3	0	10	11	4	3
Sbj 4	0	10	16	10	4
Sbj 5	0	11	17	9	4
Sbj 6	0	14	15	8	4
Sbj 7	0	21	16	7	4
Sbj 8	0	10	12	10	6
Sbj 9					
Sbj 10	0	11	7	4	2
Sbj 11	0	10	12	7	3
Sbj 12	0	9	7	8	6
M	0	11,18182	13,63636	7,454545	4,090909
SD	0	3,600505	4,610265	2,0181	1,221028

Table S1. Psilocin plasma levels during intoxication with psilocybin in the 12 final subjects. The levels were measured five times. Sbj 9 declined the blood draw. Units of measurement are ng/mol. Legend: Sbj = Subject; M = mean; SD = standard deviations

Effects of psilocybin on heart rate and blood pressure

Psilocybin led to a significant increase in systolic blood pressure at 60 minutes after intoxication, $t(11) = 6.73$, $p < 0.001$; at 120 minutes, $t(11) = 4.93$, $p < 0.001$; and normalized 4h later. Diastolic blood pressure was significantly higher at 60 minutes, $t(11) = 4$, $p = 0.002$; and 120 minutes, $t(11) = 4.847$, $p = 0.001$ after ingestion compared to the placebo. Heart rate was not significantly increased during the peak intoxication at 60 minutes compared to the placebo, $t(11) = 2.22$, $p = 0.058$; nor at 120 minutes, $t(11) = 1.32$, $p = 0.215$.

Similar results were presented in a previous study [3]; the only difference was in HR at 120 minutes, where they found a significant difference.

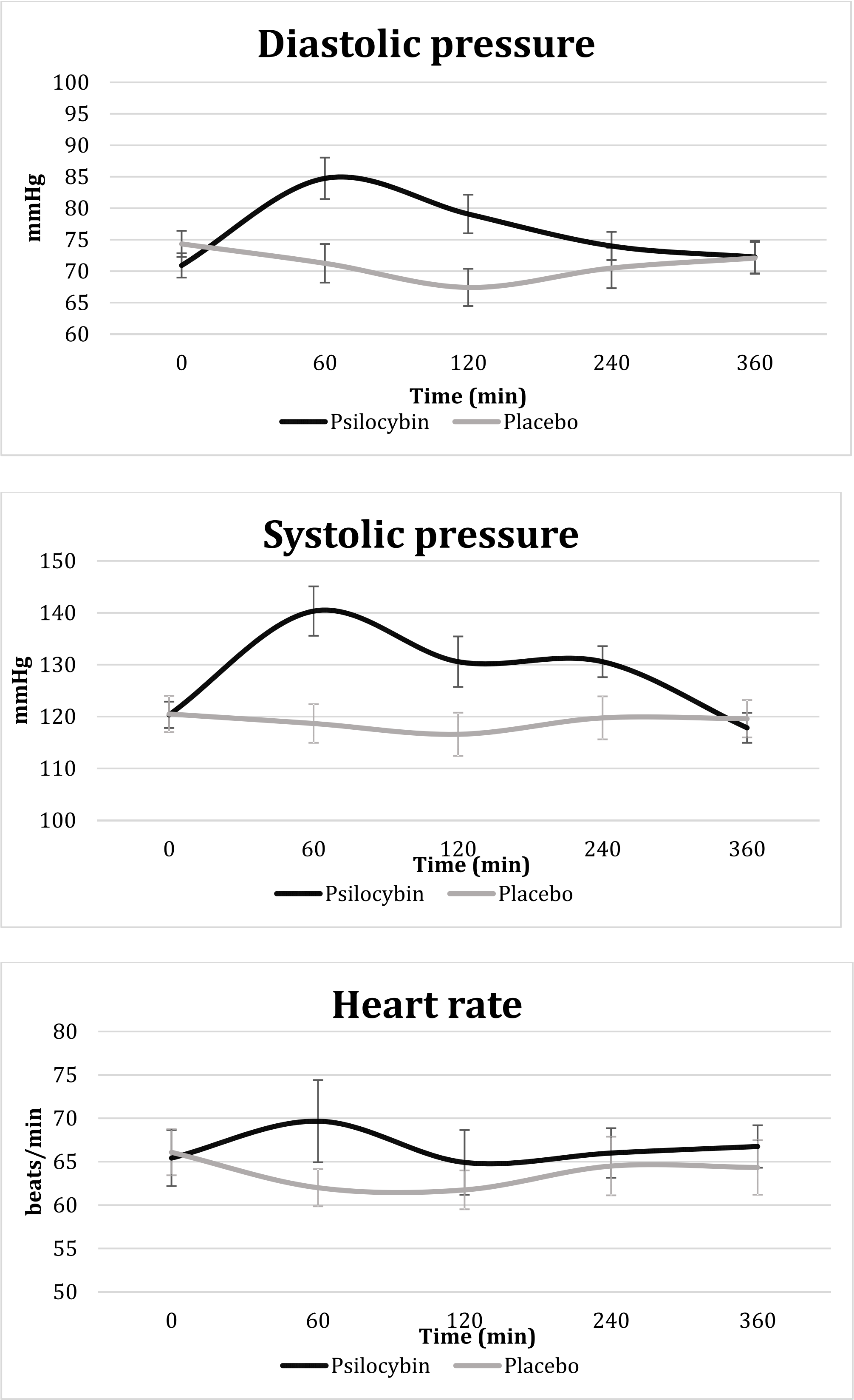

Figure S3. These graphs show the mean difference in blood pressure and heart rate during psilocybin intoxication and with placebo over time in the final sample of 12 subjects. Error lines represent standard error of the mean.

Effects of psilocybin on subjective experience and psychopathology

Analyses of the Altered states of consciousness scale revealed significant effects of psilocybin in all subscales: Experience of Unity ($t(11) = 4.074$, $p = 0.002$), Spiritual Experience ($t(11) = 4.535$, $p < 0.001$), Blissful State ($t(11) = 5.074$, $p < 0.001$), Insightfulness ($t(11) = 6.394$, $p < 0.001$), Disembodiment ($t(11) = 3.300$, $p = 0.007$), Impaired Control and Cognition ($t(11) = 5.417$, $p < 0.001$), Anxiety ($t(11) = 3.514$, $p = 0.005$), Complex Imagery ($t(11) = 10.215$, $p < 0.001$), Elementary Imagery ($t(11) = 9.015$, $p < 0.001$), Audio-Visual Synesthesiae ($t(11) = 5.030$, $p < 0.001$), and Changed Meaning of Percepts ($t(11) = 5.835$, $p < 0.001$).

Similarly, the effects of treatment were significant in almost all subscales of HRS: Somaesthaesia ($t\ (11) = 5.457$, $p < 0.001$); Affect ($t\ (11) = 4.904$, $p < 0.001$); Perception ($t\ (11) = 9.393$, $p < 0.001$); Cognition ($t\ (11) = 6.156$, $p < 0.001$); Volition ($t\ (11) = -0.911$, $p = 0.382$); and Intensity ($t\ (11) = 6.348$, $p < 0.001$). Compared to a previous study [3], we did not find significant difference in Volition, which corresponds to a change in capacity to wilfully interact with themselves, the environment, or some aspects of the experience.

A repeated measure ANOVA revealed a significant psilocybin-placebo difference in the total BPRS scores (effect of drug: $F\ (1,11) = 32.28$, $p<0.001$; drug x time: $F\ (3,33) = 12.99$, $p<0.001$), irrespective of sequence and period. Consequent pairwise comparisons indicated significantly higher total scores of BPRS 60 min, $t\ (11) = 5.348$, $p<0.001$; BPRS 175 min ($t\ (11) = 4.157$, $p = 0.002$), and BPRS 360 min ($t\ (11) = 2.209$, $p = 0.049$) during psilocybin intoxication compared to the placebo. The only significant differences between psilocybin and the placebo were found for Factor 2 ($t\ (11) = 5.380$, $p < 0.001$), Factor 3 ($t\ (11) = 5.607$, $p < 0.001$) and Factor 4 ($t\ (11) = 5.348$, $p < 0.001$) in the BPRS 60, and Factor 2 ($t\ (11) = 2.629$, $p = 0.023$), Factor 3 ($t\ (11) = 5.419$, $p < 0.001$) and Factor 4 ($t\ (11) = 3.524$, $p = 0.005$) in the BPRS 175 min. The mean values of five factors of each BPRS during the psilocybin session are shown in Fig. 6. Compared to a previous study [3], we found a significant difference in Factor 4, which corresponds to excitement and tension.

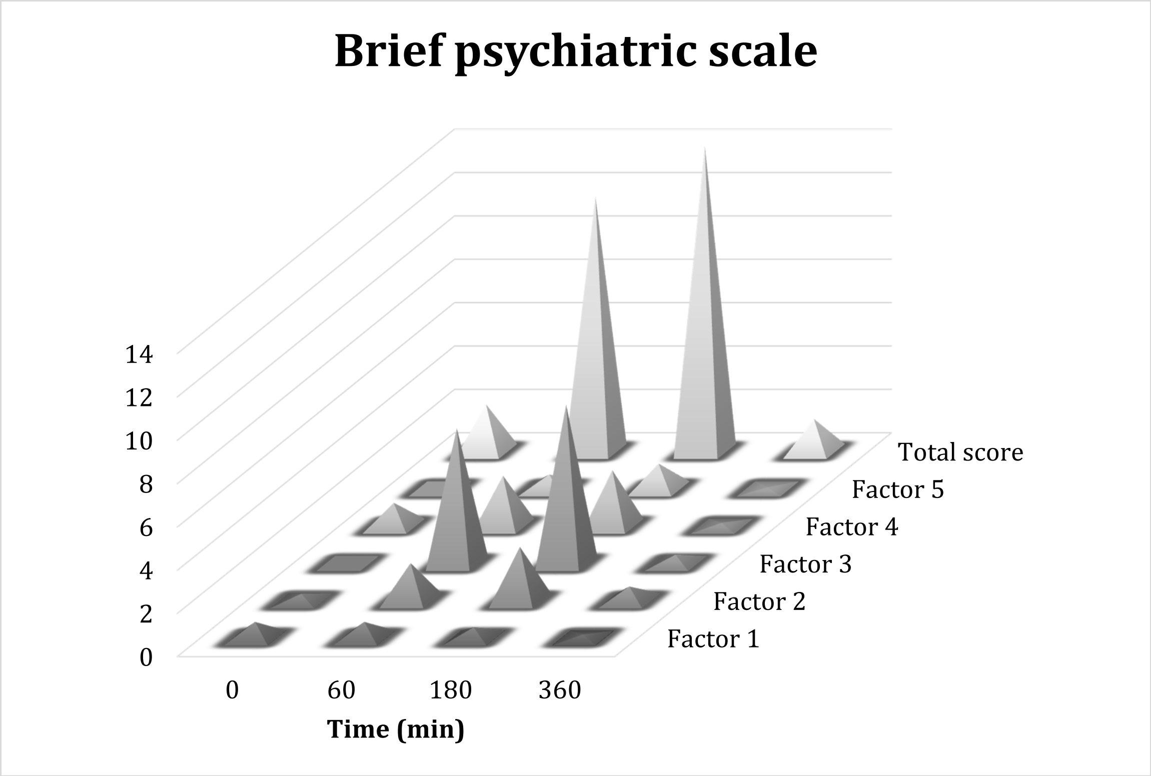

Figures S4. Mean values for Factors 1-5 of BPRS for psilocybin intoxication during baseline, at 60 and 180 min and end of session, 360 min, in 12 subjects. Factor 1: anxiety, depression, Factor 2: withdrawal, retardation, Factor 3: thought disturbance and hallucinations, Factor 4: tension, excitement, Factor 5: hostile suspiciousness.

Auditory steady-state response, subjective experience and psilocin levels correlations

		PLI		EA		ERSP	
		Mean	SD	Mean	SD	Mean	SD
Placebo	Pre	0.305	0.131	0.461	0.213	0.678	0.298
	Post	0.285	0.139	0.430	0.224	0.632	0.292
Psilocybin	Pre	0.281	0.126	0.421	0.192	0.612	0.244
	Post	0.222	0.110	0.329	0.158	0.521	0.143

Table S2. Means and standard deviations of three ASSR measures. Legend: PLI = Phase-locking index; ERSP = event-related spectral perturbation; EA = Evoked amplitude; SD = standard deviation; Pre = measurement before drug intake; Post = measurement at peak of intoxication

	PLI		ERSP		EA	
	r	p	r	p	r	p
Somaesthesia	0,434	0,158	0,209	0,515	0,344	0,273
Affect	0,628*	0,029	0,241	0,451	0,561	0,058
Perception	0,142	0,659	-0,243	0,447	0,061	0,850
Cognition	0,674*	0,016	0,332	0,291	0,605*	0,037
Volition	-0,092	0,777	0,431	0,162	-0,036	0,912
Intensity	0,552	0,063	-0,064	0,844	0,430	0,163

Table S3. Pearson correlation coefficient of HRS questionnaire and difference measures of ASSR.
* Correlation is significant at the 0.05 level (2-tailed).
Legend: PLI = Phase locking index; ERSP = Event-related spectral perturbation; EA = Evoked amplitude

	EA		ERSP		PLI	
	r	p	r	p	r	p
Somasthesia	-0,150	0,643	-0,184	0,567	-0,159	0,621
Affect	-0,171	0,595	-0,124	0,701	-0,211	0,510
Perception	-0,029	0,930	-0,100	0,756	-0,065	0,840
Cognition	-0,117	0,717	-0,135	0,676	-0,157	0,626
Volition	0,370	0,236	0,186	0,564	0,387	0,214
Intensity	-0,550	0,064	-0,455	0,137	-0,590*	0,043

Table S4. Pearson correlation coefficient of HRS questionnaire and measures of ASSR in the peak of intoxication.
* Correlation is significant at the 0.05 level (2-tailed).
Legend: PLI = Phase-locking index; ERSP = Event-related spectral perturbation; EA = Evoked amplitude

	EA		ERSP		PLI	
	r	p	r	p	r	p
OBN	0,479	0,115	0,236	0,461	0,555	0,061
DED	0,149	0,645	-0,034	0,915	0,163	0,614
VRS	-0,023	0,943	-0,319	0,312	0,079	0,808
AUA	-0,267	0,401	-0,490	0,106	-0,206	0,522
VIR	0,078	0,809	0,306	0,334	-0,061	0,851
EOU	0,364	0,244	0,300	0,343	0,445	0,148
SE	0,374	0,232	0,014	0,966	0,431	0,162
BS	0,258	0,419	0,085	0,793	0,290	0,361
IF	0,426	0,167	0,191	0,551	0,515	0,086
DB	0,262	0,411	0,062	0,848	0,377	0,227
ICC	0,343	0,275	0,009	0,977	0,363	0,247
AX	0,115	0,721	0,072	0,825	0,112	0,728
CI	0,192	0,550	-0,138	0,670	0,349	0,266
EI	-0,220	0,492	-0,535	0,073	-0,133	0,681
AVS	-0,339	0,280	-0,610	0,051	-0,247	0,439
CMP	0,100	0,756	-0,067	0,837	0,160	0,618

Table S5. Pearson correlation coefficient of 5D-ASC questionnaire and measures of ASSR.

Legend: PLI = Phase-locking index; ERSP = Event-related spectral perturbation; EA = Evoked amplitude; Experience of Unity (EOU), Spiritual Experience (SE), Blissful State (BS), Insightfulness (IF), Disembodiment (DB), Impaired Control and Cognition (ICC), Anxiety (AX), Complex Imagery (CI), Elementary Imagery (EI), Audio-Visual Synaesthesia (AVS) and Changed Meaning of Percepts (CMP)

	EA		ERSP		PLI	
	r	p	r	p	r	p
BPRS 60 min	0,299	0,345	-0,023	0,943	0,318	0,314
BPRS 175 min	0,356	0,255	-0,093	0,774	0,407	0,189

Table S6. Pearson correlation coefficient of BPRS measured in 60 and 175 minutes since administration of psilocybin and measures of ASSR.
Legend: BPRS = Brief Psychiatric Rating Scale; PLI = Phase-locking index; ERSP = Event-related spectral perturbation; EA = Evoked amplitude

	EA		ERSP		PLI	
	r	p	r	p	r	p
psilocin 120 min	-0,356	0,282	-0,048	0,889	-0,369	0,264

TABLE S7. Pearson correlation coefficient of psilocin level in 120 minutes and measures of ASSR measured in the peak of intoxication for 11 participants (one refused blood withdrawal).
Legend: PLI = Phase-locking index; ERSP = Event-related spectral perturbation; EA = Evoked amplitude

Auditory steady-state response measures correlations

	PLAC1	PLAC2	PLAC3	PLAC4
PLAC1	X	0,884**	0,863**	0,785**
PLAC2	0,884**	X	0,961**	0,756**
PSILO1	0,863**	0,961**	X	0,773**
PSILO2	0,785**	0,756**	0,773**	X

Table S8. Pearson correlation coefficient of measure of ASSR – **Evoked amplitude** at different time points.
** Correlation is significant at the 0.01 level (2-tailed).
Legend: PLAC1 = measurement before administration of placebo; PLAC2 = measurement 105 minutes after administration of placebo; PSILO1 = measurement before administration of psilocybin; PSILO2 = measurement 105 minutes after the administration of psilocybin

	PLAC1	PLAC2	PLAC3	PLAC4
PLAC1	X	0,858**	0,836**	0,797**
PLAC2	0,858**	X	0,955**	0,777**
PSILO1	0,836**	0,955**	X	0,783**
PSILO2	0,797**	0,777**	0,783**	X

Table S9. Pearson correlation coefficient of measure of ASSR – **Phase-locking index** at different time points.
** Correlation is significant at the 0.01 level (2-tailed).
Legend: PLAC1 = measurement before administration of placebo; PLAC2 = measurement 105 minutes after administration of placebo; PSILO1 = measurement before administration of psilocybin; PSILO2 = measurement 105 minutes after the administration of psilocybin

	PLAC1	PLAC2	PLAC3	PLAC4
PLAC1	X	0,855**	0,874**	0,822**
PLAC2	0,855**	X	0,889**	0,634*
PSILO1	0,874**	0,889**	X	0,783**
PSILO2	0,822**	0,634*	0,755**	X

Table S10. Pearson correlation coefficient of measure of ASSR - **Event-related spectral perturbation** at different time points.

* Correlation is significant at the 0.05 level (2-tailed).

** Correlation is significant at the 0.01 level (2-tailed).

Legend: PLAC1 = measurement before administration of placebo; PLAC2 = measurement 105 minutes after administration of placebo; PSILO1 = measurement before administration of psilocybin; PSILO2 = measurement 105 minutes after the administration of psilocybin

LITERATURE

1. Kamata, T.; Nishikawa, M.; Katagi, M.; Tsuchihashi, H. Optimized glucuronide hydrolysis for the detection of psilocin in human urine samples. *J. Chromat. B.* **2003**, *796*, 421–427. https://doi.org/10.1016/j.jchromb.2003.08.030
2. Martin, R.; Schürenkamp, J.; Pfeiffer, H.; Lehr, M.; Köhler, H. Synthesis, hydrolysis and stability of psilocin glucuronide. *Forens. Sci. Intern.* **2014**, *237*, 1–6. https://doi.org/10.1016/j.forsciint.2014.01.006
3. Bravermanová, A.; Viktorinová, M.; Tylš, F.; Novák, T.; Androvičová, R.; Korčák, J.; Horáček, J.; Balíková, M.; Griškova-Bulanova, I.; Danielová, D.; et al. Psilocybin disrupts sensory and higher order cognitive processing but not pre-attentive cognitive processing—study on P300 and mismatch negativity in healthy volunteers. *Psychopharmacology (Berl).* **2018**, *235*, 491–503. https://doi.org/10.1007/s00213-017-4807-2.
